# Loss of *MTUS1/*ATIP expression is associated with adverse outcome in advanced bladder carcinomas: data from a retrospective study

**DOI:** 10.1186/1471-2407-14-214

**Published:** 2014-03-20

**Authors:** Anja Rogler, Sabine Hoja, Johannes Giedl, Arif B Ekici, Sven Wach, Helge Taubert, Peter J Goebell, Bernd Wullich, Michael Stöckle, Jan Lehmann, Sabrina Petsch, Arndt Hartmann, Robert Stoehr

**Affiliations:** 1 Institute of Pathology, University Hospital Erlangen, Krankenhausstr. 8-10 91054 Erlangen, Germany; 2 Institute of Human Genetics, Friedrich-Alexander Universität Erlangen-Nürnberg, Schwabachanlage 10 91054 Erlangen, Germany; 3 Department of Urology, University Hospital Erlangen, Krankenhausstr. 12, 91054 Erlangen, Germany; 4 Clinic for Urology and Children’s Urology, University Hospital Saarland, Kirrberger Strasse, 66421 Homburg/Saar Germany; 5 Urology Practice Prüner Gang, Prüner Gang 15, 24105 Kiel, Germany; 6 Tumour Zentrum, Friedrich-Alexander Universität Erlangen-Nürnberg, Carl-Thiersch-Str. 7, 91052 Erlangen, Germany

**Keywords:** MTUS1, ATIP, Bladder cancer, Chromosome 8p deletions

## Abstract

**Background:**

Seventy percent of all bladder tumours tend to recur and need intensive surveillance, and a subset of tumours progress to muscle-invasive and metastatic disease. However, it is still difficult to find the adequate treatment for every individual patient as it is a very heterogeneous disease and reliable biomarkers are still missing. In our study we searched for new target genes in the critical chromosomal region 8p and investigated the potential tumour suppressor gene candidate *MTUS1/*ATIP in bladder cancer.

**Methods:**

*MTUS1* was identified to be the most promising deleted target gene at 8p in aCGH analysis with 19 papillary bladder tumours. A correlation with bladder cancer was further validated using immunohistochemistry of 85 papillary and 236 advanced bladder tumours and in functional experiments. Kaplan-Meier analysis and multivariate Cox-regression addressed overall survival (OS) and disease-specific survival (DSS) as a function of *MTUS1/*ATIP expression. Bivariate correlations investigated associations between *MTUS1/*ATIP expression, patient characteristics and histopathology. *MTUS1* expression was analysed in cell lines and overexpressed in RT112, where impact on viability, proliferation and migration was measured.

**Results:**

*MTUS1* protein expression was lost in almost 50% of all papillary and advanced bladder cancers. Survival, however, was only influenced in advanced carcinomas, where loss of *MTUS1* was associated with adverse OS and DSS. In this cohort, there was also a significant correlation of *MTUS1* expression and histological subtype: positive expression was detected in all micropapillary tumours and aberrant nuclear staining was detected in a subset of plasmocytoid urothelial carcinomas. *MTUS1* was expressed in all investigated bladder cell lines and overexpression in RT112 led to significantly decreased viability.

**Conclusions:**

*MTUS1* is a tumour suppressor gene in cultured bladder cancer cells and in advanced bladder tumours. It might represent one new target gene at chromosome 8p and can be used as an independent prognostic factor for advanced bladder cancer patients. The limitation of the study is the retrospective data analysis. Thus, findings should be validated with a prospective advanced bladder tumour cohort.

## Background

For bladder cancer, it is still difficult to predict disease progression and outcome for every individual patient as reliable biomarkers are missing. In the past few years many studies were published, which investigated new potential progression-associated factors [1-5], however prospective validation studies are needed.

For example, aberrantly methylated *TBX4* was identified as a novel potential marker for disease progression [1] and *Cathepsin E*, *Maspin*, *Plk1* and *Survivin* were proposed as new markers for progression in non-muscle-invasive bladder cancer [2]. Also an involvement of mTOR signalling pathway, as assessed by S6 protein phosphorylation, seems to be associated with increased disease recurrence, progression and worse disease specific survival [3]. Munksgaard *et al.* could identify one hitherto unknown gene, *ANXA10*, which was correlated with shorter progression-free survival when expressed at low levels [4]. Using whole exome next generation sequencing technique, Gui *et al.* were able to detect for the first time mutations in chromatin remodeling genes, like *UTX* and *MLL*, which were associated with bladder cancer [5]. Deletions on chromosome 8p are also a hallmark of bladder cancer and seem to be associated with more advanced tumour stage and increased tumour progression [6,7]. We previously found allelic loss on chromosome 8p in 25% of all investigated bladder cancers, which was significantly correlated with invasive tumour growth and with papillary growth pattern. In this context, the *SFRP1* gene was identified as one potential progression marker at 8p in bladder cancer [8].

The aim of the present study was, to identify new target genes at chromosome 8p, which are affected by chromosomal deletions and which may play a role in general tumour development, progression and outcome of bladder cancer patients.

Therefore, we analysed 9 pTa and 10 pT1 papillary bladder tumours in high-resolution array-based comparative genomic hybridization (aCGH). One promising candidate gene, *MTUS1*, was selected for further analysis.

## Methods

### Patient cohorts and tumour specimen

For aCGH analysis 9 papillary pTa and 10 papillary pT1 cryo-conserved tumours were randomly chosen from the tissue bank of the Comprehensive Cancer Center Erlangen-EMN located at the Institute of Pathology in Erlangen and DNA was isolated as described below. Tissue specimens were investigated by frozen section and all specimens contained at least 80% tumour cells.

Tissue micro arrays (TMAs) of two different bladder cancer patient cohorts were used for immunohistochemical analysis of *MTUS1*: group 1 consisted of 85 patients with non-muscle invasive (pTa or pT1) papillary tumours and group 2 of 236 patients with advanced bladder tumours (≥ pT3 and/or pN1), who all underwent radical cystectomy and received adjuvant chemotherapy. TMAs of the advanced tumour group were available at the Institute of Pathology Erlangen resulting from a previous prospective study [9], originally consisting of 327 patients. Due to tissue availability only a subgroup of 236 patients of the initial cohort was analysed. For this study IRB approval was obtained from the German Association of Urological Oncology (AUO) as well as informed written consent was obtained from all patients of participating local centers and clinics. All relevant patient characteristics and clinico- and histopathological parameters were summarized previously [9].

Papillary bladder tumours were newly assembled for this study from the tumour bank of the Comprehensive Cancer Center Erlangen-EMN located at the Institute of Pathology in Erlangen. Formalin-fixed and paraffin-embedded tumour tissues and corresponding haematoxylin-eosin stained sections were selected, tumour areas were marked and reevaluated according to histopathological stage and grade by two experienced surgical pathologists (AH, JG). Clinical Follow-up data for the papillary tumour group were obtained in collaboration with the *Tumorzentrum (TUZ) Erlangen.*

Informed written consent was obtained from all patients of the papillary tumour group as well as from aCGH tumour patients, and we obtained approval from the Clinical Ethics Committee of the University Hospital Erlangen for retrospective use of patient material in the context of the Comprehensive Cancer Center-tissue bank.

All relevant patient characteristics, histopathological data and follow-up are shown in Table 1. Additional characteristics of the advanced bladder cancer cohort, used for adjusting to multivariate Cox-regression are shown in Table 2.

**Table 1:** Patient characteristics

	**aCGH bladder tumour cohort**	**Papillary bladder tumour cohort**	**Advanced bladder tumour cohort**
Patients	n = 19	n = 85	n = 236
Age	Mean: 69.3 years	Mean: 70 years	Mean: 63 years
	Median: 68 years	Median: 71 years	Median: 63.5 years
	(± 9.9 years)	(± 11.6 years)	(± 8.4 years)
	Range: 53 – 95 years	Range: 29–97 years	Range: 38–81 years
			n.a. n = 4
Gender	Female: n = 5	Female: n = 22	Female: n = 56
	Male: n = 14	Male: n = 63	Male: n = 177
			n.a. n = 3
Stage	pTa n = 9	PUNLMP n = 1	pT1 n = 6
	pT1 n = 10	pTa n = 47	pT2 n = 29
		pT1 n = 31	pT3 n = 141
		pT2 n = 4	pT4 n = 37
		pT3 n = 1	n.a. n = 23
		pT4 n = 1	
Grade	lg n = 6	lg n = 40	G2, hg n = 28
	hg n = 13	hg n = 42	G3, hg n = 203
		n.a. = 3	n.a. n = 5
Follow-up OS	n.a.	Alive n = 65	Alive n = 76
		Dead n = 15	Dead n = 129
		n.a. n = 5	
Follow-up DSS	n.a.	Alive n = 70	Alive n = 63
		Dead n = 8	Dead n = 142
		n.a. n =7	

*Abbreviations*: *aCGH* array based comparative genomic hybridization, *OS* overall survival, *DSS* disease-specific survival.

**Table 2:** Additional characteristics of the advanced bladder cancer cohort, used for adjusting to multivariate Cox-regression

	
**Histological variant (n)**	
	
Common urothelial carcinoma	201
Plasmocytoid urothelial carcinoma	17
Micropapillary urothelial carcinoma	10
n.a.	8
**Type of adjuvant chemotherapy (n)**	
Gemcitabine-cisplatin	55
Mono gemcitabine	37
MVEC	64
Cisplatin-methotrexate	74
n.a.	6
**Lymph-node invasion (n)**	
pN0	98
pN1	45
pN2	70
pN3	1
n.a.	22
**P53 expression (n)**	
< 5%	85
≥ 5%	133
n.a.	18

*Abbreviations*: *n.a.* not available, *MVEC* methotrexate, vinblastine, epirubicine, cisplatin, *n* number.

### Cell lines and transfection

For functional analysis of *MTUS1*-expression, the bladder cancer cell lines RT112, RT4, J82 and BFTC905 [10-13] as well as the two presumably normal urothelial cell lines UROtsa and HCV29 were screened using qRT-PCR and Westernblot analysis. UROtsa was isolated from a primary culture of normal human urothelium and immortalized with a construct containing SV40 large T antigen [14]. For HCV29 various characterizations can be found in literature. Riesenberg *et al.* describes HCV29 as non-malignant cell line of the ureter region [15], whereas other groups designate it as pre-malignant or even malignant cell line [16-18]. Thus, it seems more appropriate to term these apparently normal cell lines UROtsa and HVC29 as immortal urothelial cell lines with no or low malignant potential. Cells were cultured in RPMI medium supplemented with 10% fetal calf serum (FCS), 1% sodium-pyruvate and 1% L-glutamine at 37°C and 5% CO_2_. The prostate carcinoma cell line LNCaP was used as positive control for *MTUS1*-expression [19].

Transfection was carried out in 6-well plates seeding 300 000 cells per well. After 48 hours of cell adhesion *MTUS1* was transiently overexpressed in RT112 using the *MTUS1* human cDNA clone in pCMV6-XL5 vector (Origene Technologies, Rockville/USA, SC300343, transcript variant 1 = ATIP3) and MegaTran 1.0 transfection reagent (Origene Technologies) with a ratio of 1:3 (DNA:MegaTran) according to manufacturer’s instructions.

### DNA-, RNA isolation and cDNA synthesis

To investigate 19 bladder tumours in aCGH analysis, tumour specimens were manually microdissected and DNA was isolated using the *QIAamp DNA Mini Kit* (Qiagen, Hilden/Germany) according to manufacturer’s protocol. To analyse *MTUS1* gene expression with qRT-PCR, RNA was isolated using *RNeasy® Mini Kit* (Qiagen) and cDNA was converted using the *RevertAid™H Minus First Strand cDNA Synthesis Kit* (Fermentas Life Sciences, St. Leon-Rot/Germany) according to manufacturer’s instructions. For cDNA-synthesis 1 μg total RNA was used. DNA- and RNA-quality was controlled using the Multiplate Reader *Synergy 2* (BioTek, Bad Friedrichshall/Germany).

### aCGH analysis

DNA of 19 papillary bladder tumours (500 ng each) was investigated for chromosomal alterations and copy number changes with array-based comparative genomic hybridization (aCGH) using *Genome-Wide SNP Array 6.0* (Affymetrix, Munich/Germany) according to manufacturer’s protocol. Array chips were scanned with *GeneChip Scanner 3000 7G*. Hybridization was performed at the *IZKF Z3 Core Unit Genomics* of the Institute of Human Genetics in Erlangen. Data analysis was performed with *Genotyping Console* (Affymetrix). Tumour DNAs were compared with DNAs from 167 anonymous healthy controls, which were provided by the *IZKF Z3 Core Unit Genomics*.

### qRT-PCR

To analyse *MTUS1* wildtype mRNA expression in cell lines and to control overexpression of *MTUS1* in RT112, *SYBR Green*-based quantitative real-time PCR (qRT-PCR) was performed in *7500 Fast Real-time PCR-system* (Applied Biosystems, Darmstadt/Germany) with standard thermal cycling conditions. For qRT-PCR 25 ng cDNA template, 200 nM *MTUS1*-Primermix (sense: 5′-AGCTTCGGGACACTTACATT-3′, antisense: 5′-ATAGGCCTTCTTTAGCAATTC-3′), 250nM GAPDH-primermix (sense: 5′-TGGTCACCAGGGCTGCTT-3′, antisense: 5′- AGCTTCCCGTTCTCAGCC-3′) and 6.25 μl SYBR Green Mix (2×) was used in a total volume of 12.5 μl. Data analysis was performed with *7500 Software v2.0.5* (Applied Biosystems) and gene-expression ratios were calculated with ΔΔC_T_-method [20].

### *FGFR3* mutation analysis

*FGFR3* mutation analysis was performed as previously described [21-23]. Extended primers were separated by capillary electrophoresis in the *Genetic Analyser 3500 Dx* (Applied Biosystems), and the presence or absence of a mutation was indicated by the incorporated wildtype or mutant labelled dideoxy nucleotide.

### Western blotting

To analyze *MTUS1* protein expression in cell lines, immunoblotting was performed with 30 μg total protein of whole cell lysates after SDS-PAGE on 7.5% PAA-gels on nitrocellulose membrane using wet blotting method with *Mini Protean® Tetra System* (BioRad Laboratories, Munich/Germany) according to manufacturer’s protocol. Membranes were blocked with *Immunoblot Blocking Reagent* (Millipore, Billerica/USA) and treated with anti-*MTUS1 antibody* (mouse IgG clone 1C7, Abnova H00057509-M01, 1:130, 1 hour/RT, contains epitopes against ATIP1 (49 kDa), ATIP3 (140 kDa) and ATIP4 (59 kDa)) or β-AKTIN (mouse, Sigma-Aldrich, Taufkirchen/Germany, A5441, 1:10 000, 1 hour, RT) and HRP-conjugated secondary antibody (goat-anti-mouse, Dianova/Jackson ImmunoResearch Laboratories, Baltimore/USA, 40 min, RT). Luminescence signal detection was performed using *Immobilion Western Chemiluminescent HRP Substrate* (Millipore) according to manufacturer’s instructions with *Fusion FX7* (Vilber-Lourmat, Eberhardzell/Germany). Cell lysates of LNCaP were included as positive control.

### Immunohistochemistry

Immunohistochemistry was performed on formalin-fixed, paraffin-embedded (FFPE-) 4 μm TMA sections of tumour tissue specimen transferred to glass slides. TMA construction was performed as described previously [24,25]. TMAs were stained with monoclonal mouse anti-*MTUS1* antibody (Abnova, Heidelberg/Germany, overnight, RT). This was followed by incubation with secondary rabbit anti-mouse antibody (1:100 diluted in TRIS-buffer, DakoCytomation, Glostrup/Denmark) for 30 min at room temperature. Then, slides were incubated for 20 min with ABC-solution (antibody-biotin-complex *VECTASTAIN® Elite ABC kit,* Vector Laboratories, Burlingame/USA), followed by a 10 min incubation with TSA-solution (TSA™ indirect, Perkin Elmer, Waltham/Massachusetts) and 20 min reincubation with ABC according to manufacturer’s protocols. AEC-solution (*AEC Peroxidase Substrate Kit*, Vector Laboratories) was added until staining intensity was sufficient (approx. 10 min). Slides were counterstained for 2 min with haemalaun (Carl Roth, Karlsruhe/Germany) and mounted with Aquatex (Merck, Darmstadt/Germany).

Stainings were examined and evaluated by an experienced uropathologist (AH) and immunoreactivity (IRS = immune reactive score) was scored as follows: Intensity (0 = negative, 1 = weak, 2 = moderate, 3 = strong) and number of tumour cells (in percent) was determined. Number of stained cells was correlated to numbers from 0 to 4. No staining of cells was evaluated as 0, <10% as 1, 10-50% as 2, 51-80% as 3 and 81-100% as 4. Numbers were multiplied with staining intensity and immunoreactive values between 0 and 12 were created. For *MTUS1*-staining two immunoreactive groups were created: group 1 = IRS 0, group 2 = IRS 1–12.

### Viability and proliferation assay

To investigate functional consequences of *MTUS1* overexpression, effects on viability and proliferation were analysed. Therefore 15 000 cells per well were seeded into white (viability) or clear (proliferation) 96-well plates in RPMI medium. Viability and proliferation were measured after 24 hours with *CellTiter-Glo Luminescent Cell Viability Assay* (Promega, Mannheim/Germany) and with the colorimetric *QIA58 BrdU Cell Proliferation Assay* (Merck), respectively, according to manufacturer’s protocol using the Multiplate Reader *Synergy 2* (BioTek).

### Wound-healing assay

To analyse effects on migration, wound-healing assay was performed using *Culture-Inserts for Live Cell Analysis* (Ibidi, Martinsried/Germany) and photo documentation with *Olympus IX81* (Olympus Europe Holding, Hamburg/Germany). Transfected and control cells were seeded in culture-inserts with a concentration of 500 000 cells/ml using 70 μl of cell suspension per well. After cells have grown to a dense cell layer, inserts were removed and growth pattern was documented photographically within 24 hours. Area of overgrown surface between transfected cells and controls was compared using *Axio Vision Rel 4.8.2* Software (Olympus Europe Holding).

### Statistical analysis

For statistical analysis PASW/SPSS 19.0 (IBM, Armonk/New York State) was used. To determine statistical significance of differences in functional cell culture experiments, non-parametrical Kruskal-Wallis-test (for k-independent random samples, univariate analysis of variance) was used. To determine *MTUS1*-dependant survival, Kaplan-Meier analysis was performed using Log-Rank test. Survival probability and survival risk was determined with multivariate Cox-Regression analysis (95% CI). To correlate patient data amongst each other and to detect significant associations, bivariate correlation with Spearman’s rho-test and Chi-square-test was performed. P-values <0.05 were considered as statistically significant.

## Results

### aCGH analysis

We analysed a cohort of 9 pTa and 10 pT1 papillary bladder tumours for characteristic chromosomal alterations using aCGH.

**Figure 1:**
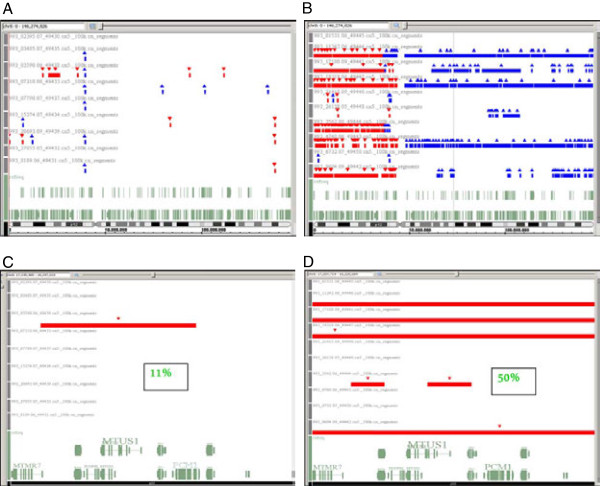
**Results of aCGH analysis from 19 papillary bladder tumours. A** Genotyping Console software depicts chromosomal alterations on chromosome 8 of all pTa, 1**B** of all pT1 tumours that were analysed. Chromosome 8 is shown in horizontal view at the bottom of the figure. Red triangles and shades stand for loss of genetic material, blue color indicates chromosomal gain. 1**C** and **D** show chromosomal loss at 8p22, where *MTUS1* is located, in 1/9 pTa tumours (1**C**) and in 5/10 pT1 tumours (1**D**).

Figure 1 depicts chromosome 8 alterations in all investigated tumours in horizontal view. In general pTa tumours (1A) had a smaller number of chromosomal alterations, than pT1 tumours (1B). In pT1 tumours there was an increased occurrence of deletions on chromosome 8p and of amplifications on 8q compared to pTa tumours. To find deleted target genes that might identify potentially progressing pTa tumours, we analysed all occurring microdeletions in pTa tumours and compared them with pT1 tumours. We found that two non-invasive tumours showed sporadic deletions on 8p. In pT1 tumours, 6/10 tumours showed almost complete loss of whole chromosome arm 8p and 2/10 tumours showed local microdeletions. Only two pT1 tumours had no detectable deletion on 8p. In Figure 1C and D, one representative microdeletion on chromosome 8p22 is shown. Only one pTa tumour (11%, 1C), but five pT1 tumours (50%, 1D) were affected by this heterozygous deletion. At this locus the following candidate target genes were identified: *SLC7a2* (*solute carrier family 7, member 2*), *PDGFRL* (*platelet-derived growth factor receptor-like*), *MTUS1* (*microtubule-associated tumour suppressor 1*), *FGL1* (*fibrinogen-like 1*) and *PCM1* (*pericentriolar material 1*). Thereof *MTUS1* was the most promising gene, as it was previously described to be a tumour suppressor gene in various malignancies, e.g. pancreatic, ovarian, colon and breast carcinomas [26-29].

As there was no known association between *MTUS1* and bladder cancer during time of analysis, we selected this gene for further characterization. In the meantime another study group also found an association between *MTUS1* expression and bladder cancer [30].

### *MTUS1* mRNA and protein expression in cell lines

**Figure 2:**
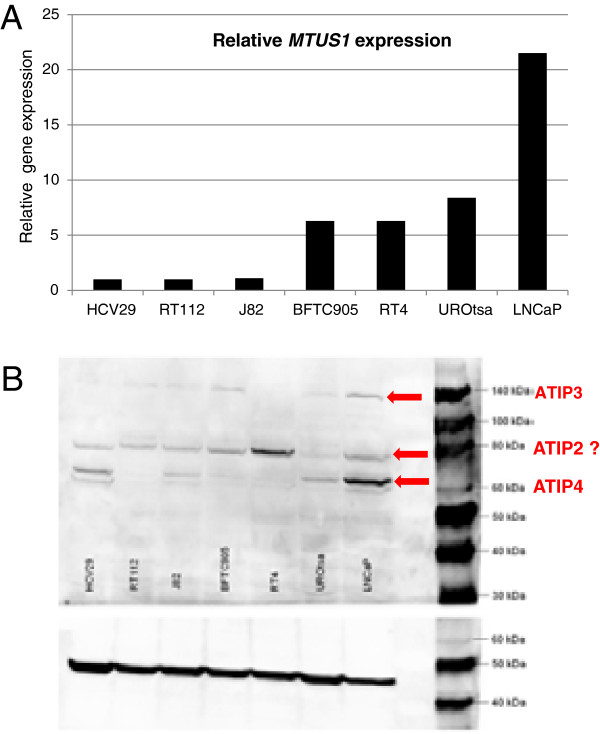
**mRNA and protein expression of *MTUS1 *in cell lines. A**. Relative quantification results after qRT-PCR indicate that *MTUS1* is expressed in all cell lines investigated. Prostate cancer cell line LNCaP, used as positive control, shows the highest expression, HCV29, RT112 and J82 the lowest. **B**. Westernblot results with anti-*MTUS1* antibody. Detection of several ATIP isoforms could be observed at 60, 80 and 140 kDa.

We screened four bladder cancer cell lines (RT112, RT4, J82 and BFTC905) as well as two presumably normal and immortalized urothelial cell lines, UROtsa and HCV29, for *MTUS1* mRNA expression using qRT-PCR. Quantitative RT-PCR analysis revealed positive *MTUS1* mRNA expression in all cell lines investigated with HCV29, RT112 and J82 having the lowest levels and UROtsa having the highest level of all bladder cell lines (Figure 2). Expression level of RT112 was defined as 100%.

These results were in line with western blotting results, where *MTUS1* bands could be detected in all cell lines analyzed (Figure 2B). However, depending on the cell type, different protein bands could be detected. The Uniprot database lists a total of 7 known protein isoforms for *MTUS1* (http://www.uniprot.org/uniprot/Q9ULD2). For RT112, J82, BFTC905, UROtsa and LNCaP (positive control) a band at around 140 kDa was visible. According to the molecular weight, this band can be attributed to *MTUS1* isoform 1 (141 kDa, ATIP3a) or isoform 2 (136 kDa, ATIP3b). A very distinct band could be observed at ~60 kDA mainly in LNCaP, HCV29 and UROtsa cells. According to the molecular weight, this band can be attributed to *MTUS1* isoform 6 (59 kDa, ATIP4). Interestingly, one additional band at approximately 80 kDA was detected in all cell lines with the strongest intensity in RT4 and the lowest in UROtsa. The origin of this band remains unknown. Although there exists a *MTUS1* isoform with a molecular weight of 84 kDa (ATIP2), this known isoform does not contain the protein epitope the antibody was raised against.

### Functional analysis after *MTUS1* overexpression in RT112

**Figure 3:**
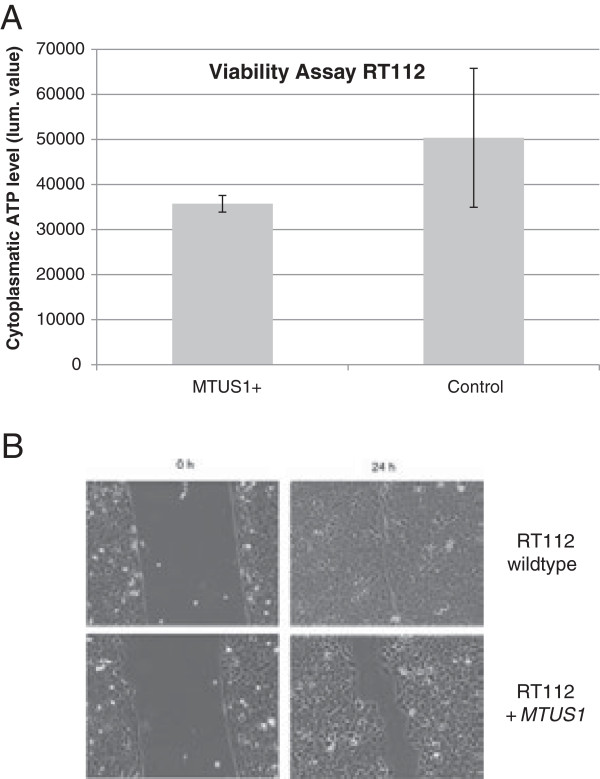
**Functional effects of *MTUS1 *overexpression.***MTUS1* overexpression in RT112 influenced cell viability (3**A**) and wound-healing (3**B**) significantly. RT112 cells with *MTUS1* overexpression showed decreased viability and retarded wound-healing after 24 hours compared to untreated control cells.

As RT112 had the lowest expression on mRNA level of all investigated bladder cancer cell lines, we selected this cell line for overexpression analysis. Therefore we transiently overexpressed *MTUS1* in RT112 and analysed effects on proliferation, viability and wound-healing after 24 hours. For proliferation, we detected an approx. 10% reduction in *MTUS1* overexpressing RT112 cells. This reduction was not statistically significant (p = 0.6, data not shown). However, it could be shown that viability was significantly decreased in *MTUS1* overexpressing cells compared to control RT112 cells (p = 0.002, Figure 3A). Regarding wound-healing assay, we found that there was a distinct but not significant difference between *MTUS1* overexpressing and wild type RT112 cells (p = 0.121). In *MTUS1*-overexpressing cells only 65.9% of the wound area was overgrown after 24 hours, whereas in wild type cells already 92.4% of the gap was closed (Figure 3B).

### IHC analysis of *MTUS1* expression in bladder tumours

**Figure 4:**
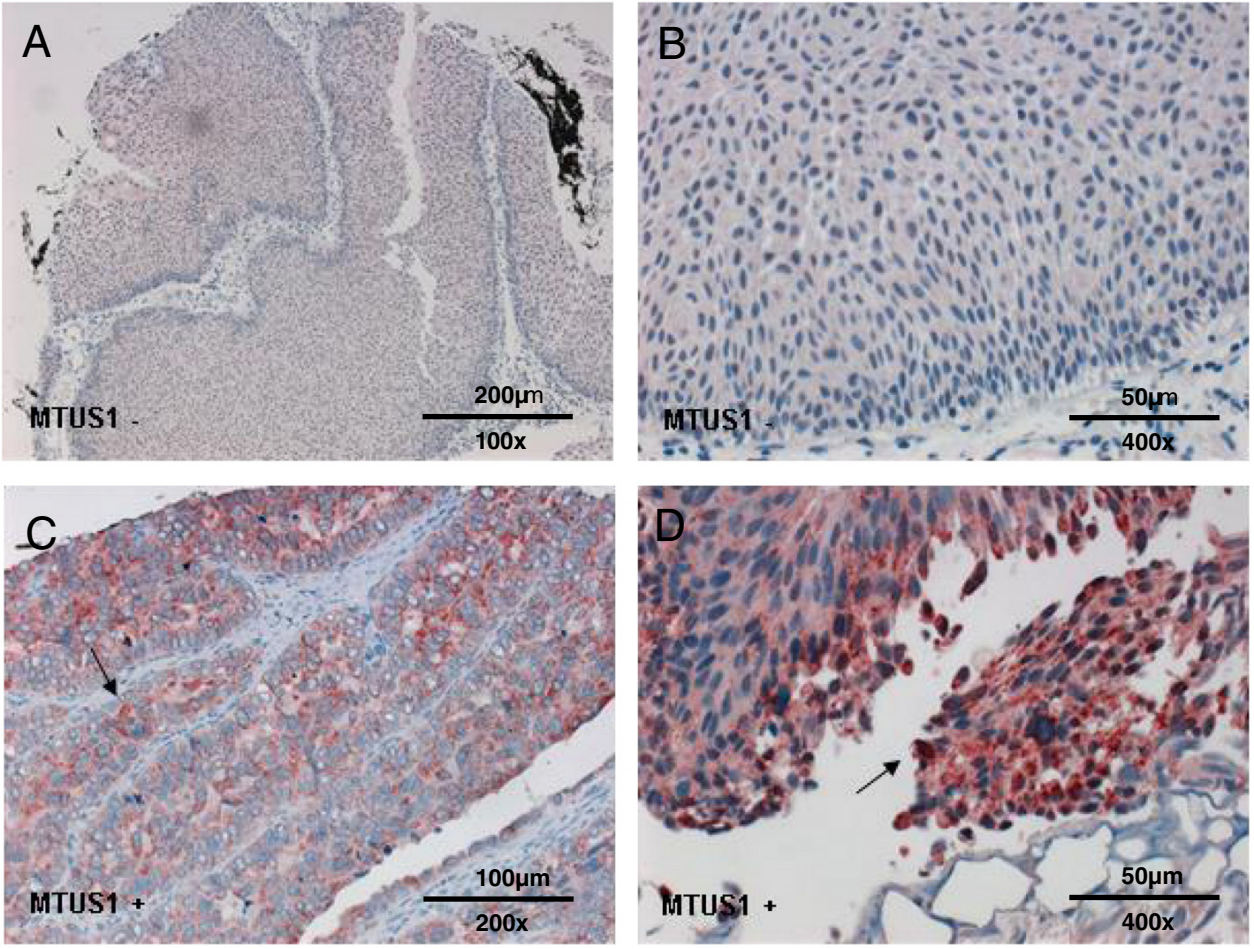
**Immunohistochemical expression of *MTUS1 *in papillary bladder tumours.** Representative examples of immunohistochemical stainings of papillary bladder carcinomas with anti-*MTUS1* antibody. 4**A** and **B** show tumours with negative, 4**C** and **D** with positive staining. Localization of *MTUS1* is in the cytoplasm.

In the papillary bladder cancer group *MTUS1* expression was lost in 50.6% of the tumours (43/85). Immunohistochemical staining showed the following distribution of immunoreactive groups: IRS 0, n = 43; IRS 2, n = 29; IRS 4, n = 8; IRS 6, n = 3; IRS 8, n = 1; IRS 9, n = 1. *MTUS1* was located in the cytoplasm, as expected. Representative examples of negative (4A, B) and positive (4C, D) staining in papillary bladder carcinomas are shown in Figure 4. Mean follow-up time was 49.5 months (median 39.1 months). Regarding overall, disease-specific, progression-free, recurrence-free and metastasis-free survival, no significant *MTUS1*-dependant differences were found.

**Figure 5:**
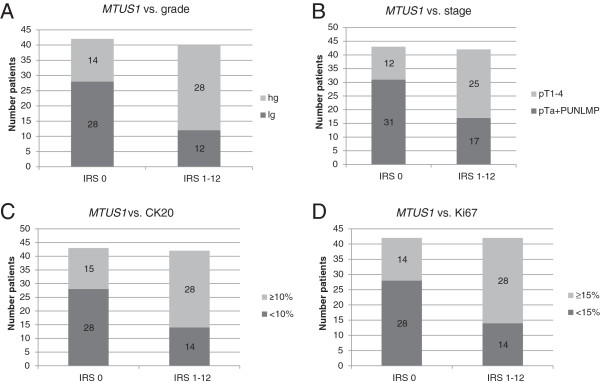
**Associations of *MTUS1 *loss with histopathological parameters in papillary bladder tumours.** Significant direct correlation of *MTUS1* expression with tumour grade (5**A**), stage (5**B**), CK20 (5**C**) and Ki67 (5**D**) expression in papillary bladder tumours. *MTUS1* loss was associated with decreased malignant potential of the cell as indicated through increased occurrence of low-grade differentiation, non-invasive growth pattern and weak CK20 and Ki67 expression in carcinomas of the IRS0 (*MTUS1*-deficient) group.

**Figure 6:**
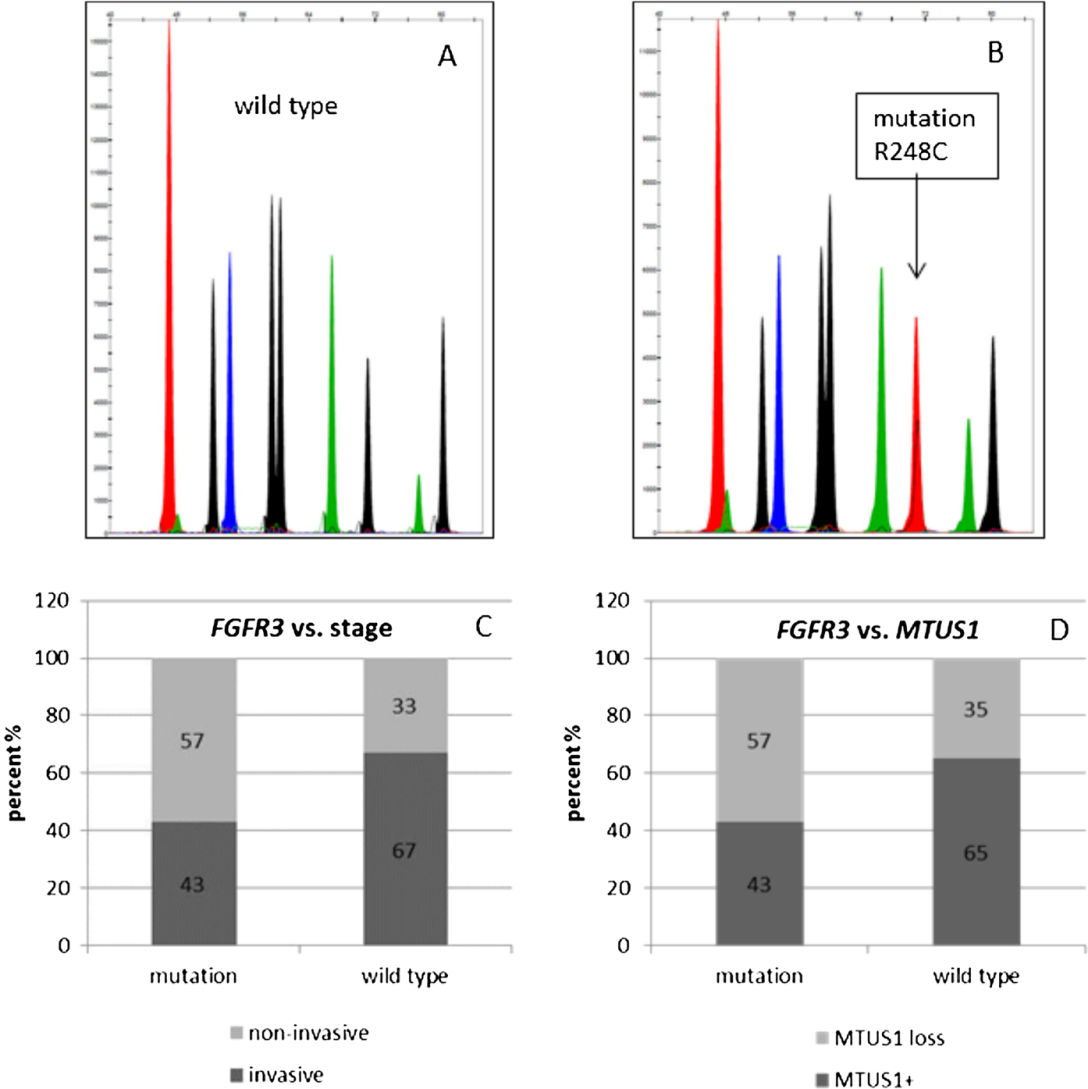
**Association of *MTUS1 *loss with *FGFR3 *mutations in papillary bladder tumours.***FGFR3* mutation analysis in the papillary bladder tumour group. 6**A** and **B** show representative examples of *FGFR3* wildtype (6**A**) and mutation R248C (6**B**) sequences. To assess data integrity we correlated *FGFR3* status with tumour stage (6**C**) and then with *MTUS1* expression (6**D**). We showed that the majority of *FGFR3* mutation tumours had a predominantly non-invasive growth pattern compared to *FGFR3* wildtype tumours, which showed more invasive phenotypes. *FGFR3* mutation where, however, correlated with *MTUS1* loss.

Interestingly a significant direct correlation between *MTUS1*-expression and stage, grade, Ki67 and CK20-expression was found (Figure 5). *MTUS1*-expressing tumours showed higher tumour grade (p = 0.005, 5A) and stage (p = 0.004, 5B) as well as aberrant expression of differentiation marker CK20 (p = 0.004, 5C) and proliferation marker Ki67 (p = 0.004, 5D). To prove the integrity of our papillary study cohort, we additionally performed *FGFR3* mutation analysis (representative examples SNaPshot analysis are shown in Figure 6A and B). It is well-known that *FGFR3* mutations occur predominantly in bladder tumours with papillary growth pattern. Those mutations are connected with a lower malignant potential of the bladder tumour as indicated by lower tumour stage and/or grade. Therefore we correlated *FGFR3* mutation status with tumour grade. The mutation analysis revealed that the majority of our tumours (n = 54) had at least one mutation, which was associated with non-invasive growth pattern, whereas only 27/85 tumours had *FGFR3* wild type (correlated with more invasive growth, p = 0.059, Figure 6C). Four tumours could not be analysed in *FGFR3* mutation analysis. Tumours with *FGFR3* mutations showed more *MTUS1* expression loss than wild type tumours (Figure 6D). This was in line with the inverse correlations of the other histopathological parameters, as mentioned above.

**Figure 7:**
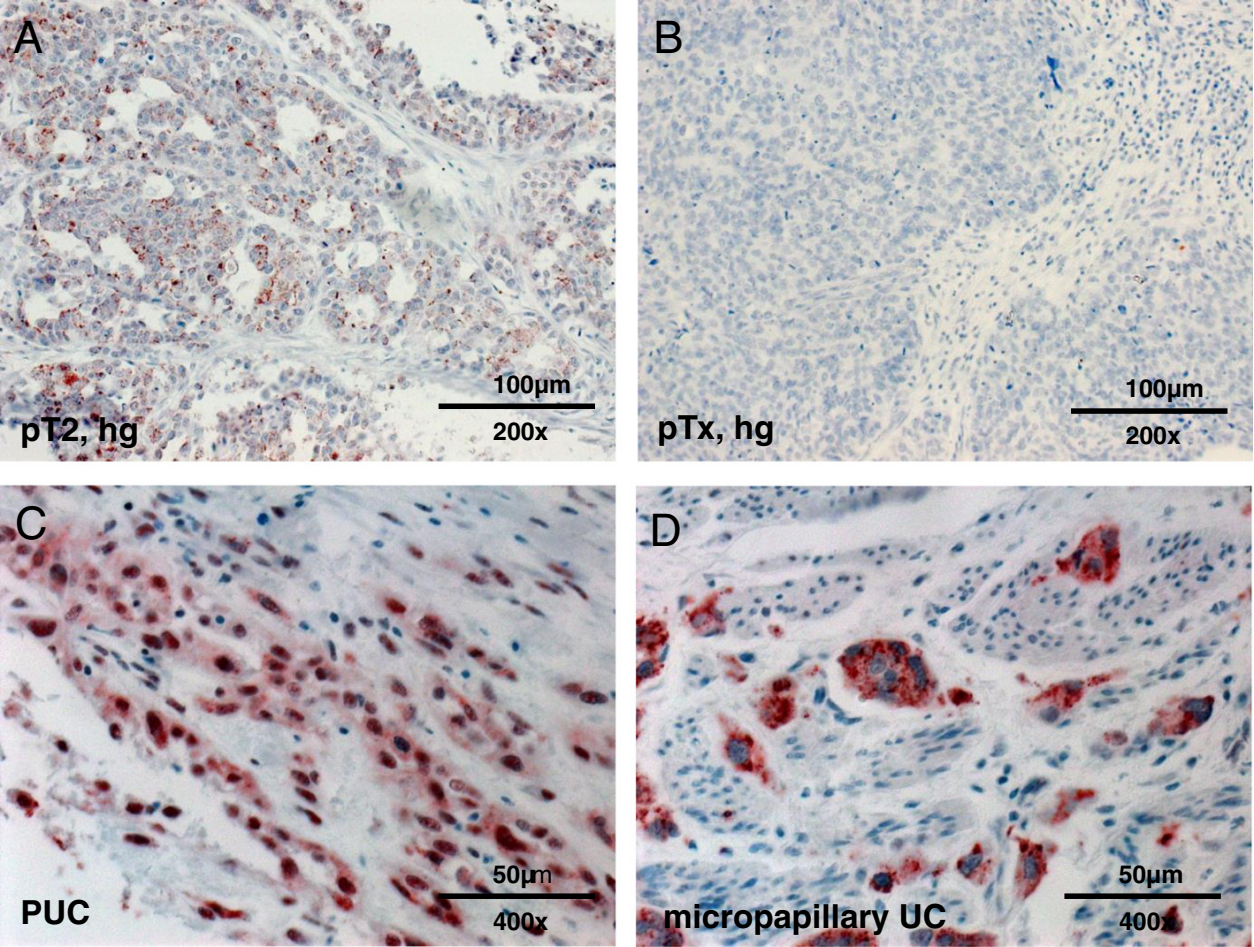
**Immunohistochemical expression of *MTUS1 *in advanced bladder tumours.** Representative examples of immunohistochemical stainings in advanced bladder tumours. 7**A** shows positive, 7**B** negative staining in common urothelial carcinoma. In 7**C** positive nuclear staining in plasmocytoid carcinoma of the bladder is shown. In all micropapillary urothelial carcinomas positive cytoplasmatic staining could be observed (7**D**).

In the advanced bladder tumour cohort 45.8% of the tumours (108/236) showed loss of *MTUS1* expression and 54% (128/236) tumours were classified into IRS group 1–12. Immunoreactivity showed the following distribution: IRS 0, n = 108; IRS 2, n = 52; IRS 3, n = 2; IRS 4, n = 43; IRS 6, n = 18; IRS 8, n = 5; IRS 9, n = 1 and IRS 12 n = 7). Representative examples of positive and negative staining of conventional advanced bladder carcinoma are shown in Figure 7A and B. Regarding the distribution of *MTUS1* expression within different histopathological subtypes (divided into common urothelial carcinoma (UC), plasmocytoid UC (PUC) and micropapillary UC), a significant expression difference was found (p = 0.011). In all micropapillary tumours (n = 10) strong positive *MTUS1*-expression was observed in the cytoplasm (Figure 7C). It was also striking that 8/17 PUCs showed positive *MTUS1* expression in the nucleus (Figure 7D), but not in the cytoplasm. The remaining PUCs showed complete loss of expression.

**Figure 8:**
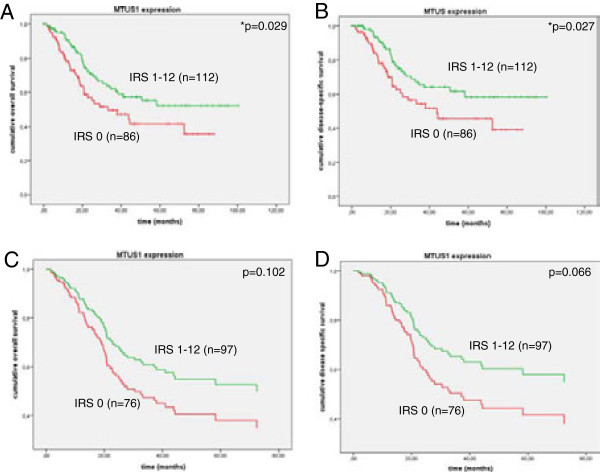
**Survival curve analysis in the advanced tumour cohort.** 8**A** and **B** show Kaplan-Meier curves for overall (8**A**) and disease specific (8**B**) survival. Patients with positive *MTUS1* expression in the tumour (green) have better overall- and disease specific survival, than tumours with *MTUS1* expression loss (red). Multivariate Cox-regression analysis (stepwise backward exclusion) for overall (8**C**) and disease-specific survival (8**D**) confirmed Kaplan-Meier findings.

Kaplan-Meier analysis revealed significantly better overall (p = 0.029) and disease-specific (p = 0.027) survival for patients with *MTUS1* expression in the bladder tumour (IRS 1–12). Patients with *MTUS1* expression survived for 64 months (OS) and 69 months (DSS), whereas patients without *MTUS1* expression showed mean survival of only 46 (OS) and 50 months (DSS), respectively. Also in multivariate Cox-regression analysis with stepwise backward elimination (adjusted to gender, stage, grade, node-invasion, histological subtype, type of chemotherapy and *P53* expression), this observation could be confirmed, however not significantly. A hazard ration of 1.507 (95% CI 0.92-2.46, p = 0.102) and 1.662 (95% CI 0.97-2.85, p = 0.066) was found for overall and disease-specific survival, respectively. OS and DSS survival curves are shown in Figure 8A to D. Due to availability of survival time and/or status as well as of additional patient characteristics, only 198/236 and 173/236 patients could be analysed in Kaplan-Meier and Cox-regression analysis, respectively. Patients with *MTUS1* expressing tumours also had better progression-free survival (PFS, mean survival time: 60.5 months, n = 111) compared to patients with *MTUS1* expression loss (46.8 months, n = 86, p = 0.179, PFS graphs not shown).

## Discussion

In aCGH we found that pT1 tumours had more genomic aberrations than pTa tumours, which strengthens the hypothesis that bladder tumours accumulate genetic alterations with progression of disease. Regarding chromosome 8p, our results were in line with previous studies, which reported loss of chromosome 8p as a common event in urothelial carcinomas [31-33]. Our most promising candidate gene identified in aCGH at 8p22, *MTUS1,* is known to be downregulated in other cancer entities, such as pancreatic, ovarian, colon, breast and prostate cancer [19,26-29]. To clarify its role in bladder cancer, we further analysed *MTUS1* in cell culture and immunohistochemical experiments.

*MTUS1* (mitochondrial or microtubulus-associated tumour suppressor 1) is located at chromosome 8p21.3-22 (17.501.304-17.658.426, NCBI Genbank ID 57509) and spans 157 kbp (including UTRs) and 110 kbp (coding region, UCSC Genome browser, uc003wxv.3) including 17 exons. Use of alternative exons leads to transcription of 30 different mRNAs and Uniprot describes seven functional *MTUS1* protein isoforms which are summarized in Table 3. The gene products are designated as ATIPs (angiotensin II AT2 receptor-interacting proteins) or as ATBPs (AT2-receptor binding-protein) and the name is derived from their function as interaction-partners of AT2-receptors of the renin-angiotensin-aldosterone system. Here ATIP mediates AT2-receptor activation and inhibition of AT1 receptor activity. As antagonist of the AT1 receptor, the AT2 receptor, enhanced through binding of ATIP*,* induces anti-proliferative and anti-apoptotic effects [34]. All ATIPs share one large C-terminal coiled-coil domain, which enables homo- and hetero-dimerization as well as their interaction with the AT2 receptor. The ATIP-proteins interact with the C-terminus of the receptor and further support its capability to inhibit *ERK2*-activity of the classical MAP-kinase-signalling pathway as well as inhibition of growth factor-induced autophosphorylation of receptor tyrosine kinases [35]. Additionally, it could be demonstrated that ATIP*3* is located at the centrosome of the cell and plays an important role in microtubulus-dynamics and mitosis. Overexpression of ATIP*3* led to extension of metaphase through modulation of the spindle-checkpoint signalling pathway and is considered as one potential therapeutic effector in metastatic breast cancer [36]. This biological function of *MTUS1/*ATIP might be also one explanation for the decreased viability in RT112 bladder cancer cells after overexpression of *MTUS1*. The distinct but not significant reduction of wound-healing behavior might be a consequence of reduced viability.

**Table 3:** Summary of ATIP isoforms and their associated transcripts and proteins (Uniprot, Q9ULD2)

***MTUS1*-Isoforms**	**Protein-variant**	**mRNA**	**Protein**	**Molecular weight**
1	ATIP3a	6435 bp	1270 aa	141 kDa
2	ATIP3b	6273 bp	1216 aa	136 kDa
3	ATIP1	3819 bp	436 aa	51 kDa
4	?	3160 bp	342 aa	38 kDa
5	ATIP2	2787 bp	770 aa	84 kDa
6	ATIP4	4022 bp	517 aa	59 kDa
7	?	2667 bp	415 aa	48 kDa

*Abbreviations*: *ATIP* Angiotensin II AT2 receptor-interacting protein, *bp* base pairs, *aa* amino acids, *kDa* kilo Dalton.

*MTUS1* was first described as a tumour suppressor gene in a study from Seibold *et al.*[26] where its function was investigated in pancreatic carcinoma cell lines as well as in several normal tissues. It could be shown that *MTUS1* was expressed in all investigated normal tissues, such as heart muscle, brain or kidney.

*MTUS1* isoforms can be classified into five groups of ATIPs: ATIP*1* (436aa, 51 kDa), ATIP*2* (770aa, 84 kDa), ATIP*3a* and *b* (1270aa, 141 kDa and 1216aa, 136 kDa) and ATIP*4* (517aa, 59 kDa). Those transcripts show an unequal distribution in human tissue. ATIP*3a* and *b* seem to be the most common variants and they can be found in almost all human tissues. ATIP3 is also designated as canonical *MTUS1* protein variant and is the predominant form reported to be expressed in the bladder [37]. Therefore, ATIP3 was used for overexpression in RT112. ATIP*1* and *4* are the predominant forms in the brain. About the distribution of ATIP*2* in human tissue not much information is available to date [37]. According to our western blot results it seems likely that, depending on the cell line, the ATIP variants 3 (~140 kDa) and 4 (~59 kDa) are expressed in bladder cancer cell lines in different concentrations. ATIP1 (49 kDa), however, seems not to be expressed in bladder cancer cell lines at all. The western blot also shows one distinct band at ~80 kDa. According to Uniprot the *MTUS1* isoform ATIP2 has a molecular weight of approximately 80 kDa. However the antibody contains no epitope for this isoform: the origin of the 80 kDa band still remains unclear. In future experiments it would be important to distinguish the expression levels of each ATIP protein separately, e.g. by usage of ATIP isoform-specific antibodies.

In immunohistochemical analysis we found that *MTUS1* expression was lost in 50.6% of all papillary and in 45.8% of all advanced bladder tumours. This loss might be the result of chromosomal deletions at 8p22, as shown in aCGH. Also epigenetic changes, like binding of microRNAs or promoter hypermethylation might inhibit gene transcription and thus protein expression. In papillary bladder cancers, survival was not influenced, however a direct correlation with stage, grade, Ki67 and CK20 expression was found. This indicates that papillary tumours with retained *MTUS1* expression have higher malignant potential than *MTUS1*-deficient tumours and that *MTUS1* should be considered more as an oncogene rather than a tumour suppressor gene. However, *MTUS1* expression did not influence survival and thus does not seem to be important for prognosis or disease progression in the papillary pathway of bladder cancer development. Our findings regarding papillary tumours make it very likely that *MTUS1* does not act as a classical tumour suppressor and make a role as new potential progression marker in papillary bladder cancer very unlikely.

Although we could find complete loss of *MTUS1* protein expression in almost 50% of the cases in both bladder tumour cohorts, survival was only influenced in the advanced bladder cancer group. Here expression loss was associated with worse OS and DSS, indicating that *MTUS1* acts as a classical tumour suppressor gene and that it might be a new target gene at chromosome 8p as well as an independent prognostic factor in advanced bladder cancer. These data argue that *MTUS1* loss could be important in the development of non-papillary bladder cancer from CIS, which should be investigated in further experiments. It might also be likely that *MTUS1* acts as a chemotherapy-response-predictor, as all investigated patients underwent chemotherapy. Additionally, *MTUS1* appears to play a major role in two variants of rare advanced and very aggressive bladder tumours. In plasmocytoid urothelial carcinomas *MTUS1* was either found in the nucleus or no expression was detected. In micropapillary tumours only positive *MTUS1* expression was found, which, in this entity, cannot be responsible for decreased malignancy, as this variant is one of the most aggressive tumour types found in the bladder. It would be interesting to clarify the biological function of *MTUS1* especially in PUCs and in micropapillary carcinomas, particularly in regard to the occurrence of mutations. One study identified five major nucleotide substitutions in ATIP3 exons in hepatocellular carcinoma [38]. For bladder cancer, however, no mutation analysis data for *MTUS1* is available yet.

In addition to our findings, one recently released study found a correlation of reduced *MTUS1* mRNA expression with poor prognosis in bladder cancer patients [30]. The patient cohort, however, was more heterogeneous than ours and comprised all kinds of transitional cell carcinomas of the bladder, ranging from pTa to pT4 and including also CIS. This study revealed equally, that *MTUS1* is an independent prognostic factor for DSS in bladder cancer.

## Conclusions

In summary, *MTUS1/*ATIP was identified as a tumor suppressor gene in cultured bladder cancer cells and in patients with advanced bladder cancers. Although *MTUS1/*ATIP loss was detected in approximately 50% of all investigated bladder cancers, there was only a significant association with worse OS and DSS in advanced bladder carcinomas, but not in papillary bladder carcinomas. This might be due to two different molecular pathways that lead to the development of either frequently recurring papillary or highly malignant solid bladder cancers. In future experiments we want to determine the expression level of potential *MTUS1*-binding microRNAs and analyse promoter methylation and mutation status of *MTUS1* in bladder tumour specimen. We further want to reveal the reason for the frequent loss of *MTUS1/*ATIP in bladder cancer and the differences between papillary, micropapillary and other advanced bladder cancers.

## Abbreviations

ATIP: Angiotensin II AT2 receptor interacting protein; MTUS1: Microtubulus-associated tumour suppressor 1; OS: Overall survival; DSS: Disease specific survival; aCGH: Array comparative genomic hybridisation; aa: Amino acid(s); bp: Basepair(s); Da: Dalton; μ: Micro; n: Nano; s: Second(s); min: Minute(s); h: Hour(s); °C: Degree(s) celsius.

## Competing interests

The author’s declare that they have no conflict of interest.

## Authors’ contributions

AR coordinated development of papillary bladder tumour tissue micro array, performed DNA- and RNA-isolation, cell culture experiments, qRT-PCR, statistical analysis, data interpretation and aCGH data analysis and participated in immunohistochemical staining, study conception and drafted the manuscript. SH performed immunohistochemical staining and analysis and participated in cell culture experiments, qRT-PCR, statistical analysis, data interpretation and aCGH data analysis. JG participated in histological evaluation of papillary bladder tumours. AE carried out aCGH analysis within the IZKF core unit *Z3 Affymetrix-Chip-Analysen.* SW participated in survival curve generation and analysis and provided LNCaP positiv control cell line and critically revised the manuscript. HT participated in Kaplan-Meier and statistical analysis and critically revised the manuscript. PG participated in study design, helped to acquire patient data. BW participated in study design, helped to acquire patient data. MS was the principle investigator of the AUO trial and provided paraffine blocks for the advanced bladder cancers (advanced TMA cohort). JL was conducting patient data requisition of the AUO trial (advanced TMA cohort). SP helped to acquire patient data for the papillary TMA cohort. AH helped to draft the manuscript, participated in study design and histological evaluation and supervised the study. RS conceived of, coordinated and supervised the study, participated in TMA development and helped to draft the manuscript. All authors read and approved the final manuscript.

## Pre-publication history

The pre-publication history for this paper can be accessed here:

http://www.biomedcentral.com/1471-2407/14/214/prepub
